# Case of Rupture of the Uterus

**Published:** 1851-05

**Authors:** Thomas J. Gallaher


*Case of Rupture of the Uterus.* By THOMAS J. GALLAHER, M. D. (Read before the Young Men's Medical Association of Pittsburgh, Feb. 5th, 1851.)

Mrs. F., a hearty, robust, and healthy Irish woman, aged 30 years, was taken in labor, with her first child, on Friday, January 24th, 1851. In a short time the membranes gave way, when a large quantity of the liquor amnii escaped from the uterus, while the patient was yet walking about the floor. Her husband and some other men came into the house about this time, which frightened her so much that her pains entirely ceased. On the following day, at one o'clock, P. M., her pains returned, when a midwife was sent for, who, upon an examination per vaginam, pronounced it a case of breech presentation, and on that account refused to take charge of it. At four o'clock, P. M., Dr. Cahill was called to the woman. He found the uterine contractions regular in their recurrence, and of usual frequency and intensity. The right elbow of the fœtus was felt protruding through the superior strait. At this time the liquor amnii had been drawn off twenty-eight hours, in consequence of which, the walls of the uterus were so firmly contracted around the body of the child as to make it impossible to turn. No attempts of the kind were, therefore, made; the Dr. contenting himself with holding the arm to one side, so that if a favorable change should take place in the position of the child, the head might descend. This expectation, however, was not realized, as about 9 o'clock, the right hand descended through the os externum. Between eleven and twelve o'clock the woman had a few pains more severe than the rest, during which she shrieked, and said she felt something tear within her, after which she had no more.

Finding the woman no nearer delivery than when first called in, and noticing the occurrences of alarming symptoms, the Dr. now requested consultation. Dr. Draine was accordingly sent for. Dr. D. remained with her till three o'clock in the morning, in company with Dr. C. Shortly after the occurrence of the severe tearing pains, above mentioned, the contractions of the uterus entirely ceased. At this period the protruded arm was pushed without any difficulty above the superior strait, and laid along the side of the child. No hemorrhage had occurred to depress the patient, while she evidently was sinking, from some cause not known to the medical gentlemen present. At this juncture it was concluded to have further medical aid. Accordingly, between three and four o'clock, in the morning, I received a polite note from Dr. Draine, requesting my assistance in the case. On arriving, I learned the history of the case, as above given,and found the woman in a most deplorable condition. Her extremities were deathly cold, her pulse was imperceptible at the wrist, and the contraction of the womb had ceased for several hours. She threw up the contents of her stomach while I was sitting at her bed-side, and appeared in great agony, tossing about the bed.

Passing my finger into the vagina, I found the placenta lying loosely within the os externum, which I removed without difficulty. Finding the cord pulseless I cut it, when no blood flowed from its cut extremities. From this I well knew that the child was dead. On passing my hand up the vagina, I felt the right shoulder of the child, presenting at the superior strait, with its head in the left iliac fossa, and its back towards the abdomen of the mother. To the left of the child was a thick, rough and hard fleshy body, larger than my hand, which I could move slightly about. At this time I was unable to conjecture what this substance could be. The child could be moved about with great ease, and its vertex was brought to the superior rim of the pelvis without any difficulty, but then there was no contraction of the uterus to make it descend. My hand was then pushed higher up on the body of the child, when, to my surprise, it was encircled by the intestines of the mother. The conviction immediately flashed upon me that the uterus was ruptured, and that the child had escaped from its cavity to that of the abdomen. Examining the case still further, I found the child surrounded by intestines and omentum, while I was convinced that the rough, fleshy body already described, and lying on the left side of the mother, was the body of the uterus in a collapsed state. I could not ascertain how much of the uterus was implicated in the accident, but the whole superior part of the vagina, excepting some on the left side, was torn from its connection with that organ. The laceration was so extensive and gaping, that on the first introduction of the hand it was difficult to detect it.

On withdrawing my hand I informed the medical gentlemen present as to the condition of the uterus and vagina, and the situation of the child, and expressed my conviction that it was entirely unnecessary to deliver the woman, as she would assuredly die whether delivered or not, and the child was already dead. All efforts of the kind would merely give her unnecessary pain, without contributing to save her life. I insisted it was better to let her die in peace than to give her unnecessary torture. After some conversation on the subject, it was decided to abandon the unhappy sufferer to her fate.

Between eight and nine o'clock in the morning, Dr. M'Neal visited with us the patient, and fully concurred in the diagnosis and prognosis of the case which we had made. He likewise concurred in the opinion that it was unnecessary to deliver her.

After suffering great pain from the supervention of fatal peritonitis, the patient died between six and seven o'clock the same evening. She lived at least nineteen hours after the uterus had been ruptured, and fifteen after the pulse had ceased to beat, and her extremities began to be cold.

No post-mortem examination could be obtained.

*Remarks.*—In most cases of rupture of the uterus, the organ is found in a morbid condition, but whether, in this case, there was disease, could not be ascertained. The inference to be drawn from the previously healthy condition of the woman, and the circumstances of the case, is, that the womb was not diseased before the commencement of labor. The cause of the rupture must therefore be explained on other grounds.

The presentation of the shoulder was no doubt the *predisposing*, whilst the frequent, powerful and long-continued contractions of the uterine fibres around the body of the fœtus when it was immoveably fixed in the passages, were the *immediate* cause of the casualty.

Some one might ask, why not deliver at once when the nature of the accident was first discovered? I answer, had we seen the patient early, and detected the nature of the complication in time, it would have been our imperious duty to turn and deliver at once. This would perhaps have given the woman the only chance, slim as it was, of life. But why attempt to deliver her when the fœtus is dead and the woman dying? It certainly would have imposed on the mother unnecessary pain. Another view of the case is,—Had we delivered the dying woman, and had she died shortly afterwards, as unquestionably she would, her friends, and the clamoring multitude always found in such places, would have blamed her death, and perhaps the child's, upon us, and trumpeted it to the world. Therefore, for the comfort of the woman, and the safety of our own reputation, we think we acted the proper part in abstaining from any unnecessary interference.

[From the conclusions of our correspondent we feel compelled to dissent. It appears to us that the nature of the accident was evident sufficiently early to give the patient a chance for her life, since shortly after its occurrence the arm was pushed up and laid along the side of the child, and when seen by Dr. G., the body could be moved, and the vertex brought to the superior strait. Surely, at this time, the woman might have been delivered by version by the feet, and not left to her fate. Under such circumstances, it is even recommended, by high authorities, to perform the abdominal section, rather than let the woman die undelivered. In all cases where it is possible, the testimony is in favor of early delivery; and the cases of recovery confirm this decision, for in all but one or two, the women were delivered. In confirmation of the opinion which we thus advance in all kindness and respect to our correspondent, we cite the authority of Ramsbotham, Burns, Dewees, John Clark, Collins, Velpeau, Meigs, Siebold, Baudelocque, Boivin, Churchill, and others, many of whom express themselves in favor of delivery even when the accident has been of some hours duration, or the woman exhausted by constitutional shock and irritation. "The properest indication must always be to extract the child."

As regards the second reason for non-interference—to wit: the clamoring of the multitude—we can only say, "*fiat justitia, ruat cœlum.*"—Eds. Examiner.]

